# Supplementary material for: Polymethoxylated Flavones Target Cancer Stemness and Improve the Antiproliferative Effect of 5-Fluorouracil in a 3D Cell Model of Colorectal Cancer
Source: Nutrients. 2019 Feb 2;11(2):326. doi: 10.3390/nu11020326 (PMC6412836; doi:10.3390/nu11020326)
Supplement: Supplementary file 1 [file nutrients-11-00326-s001.pdf]

Supplementary Material

Polymethoxylated flavones target cancer stemness and improve the antiproliferative effect of 5-Fluorouracil in a 3D cell model of colorectal cancer

Carolina V. Pereira, Marlene Duarte, Patrícia Silva, Andreia Bento da Silva, Catarina M. M. Duarte, Alejandro Cifuentes, VirginiaGarcía-Cañas, Maria R. Bronze, Cristina Albuquerque and Ana Teresa Serra

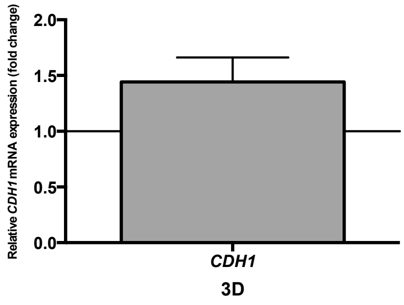

Figure S1. Characterization of 3D cell model (HT29 spheroids) on day 7 of culture. Relative mRNA expression of epithelial marker *CDH1* (encoding E-cadherin) by qPCR. Results were normalized relative to the HT29 monolayer cells and expressed as mean ± SD of three independent experiments.

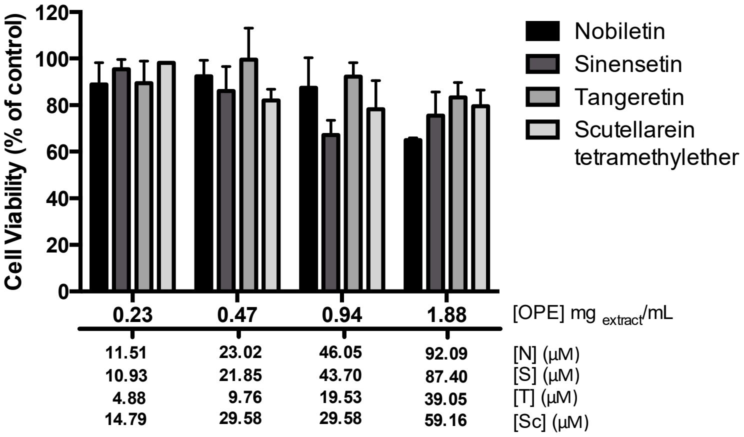

Figure S2. Antiproliferative effect polymethoxylated flavones (PMFs) in HT29 cell spheroid proliferation tested in the same concentration as in the extract (72 hours). Data are mean ± SD of four independent experiments performed with six replicates. Legend: N—nobiletin; S—sinensetin; T—tangeretin; Sc—scutellarein tetramethylether.

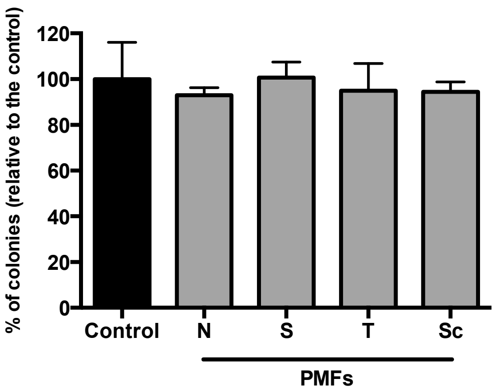

Figure S3. Inhibitory effect of PMFs in anchorage-independent cell growth using cells derived from HT29 spheroids. Inhibition of colony formation by PMFs in equivalent concentration present in 0.35 mg/mL of OPE (nobiletin—17.11 μM, sinensetin—16.24 μM, tangeretin—3.63 μM, and scutellarein tetramethylether—10.99 μM). All data are expressed mean ± SD of at least three independent experiments. Legend: N—nobiletin; S—sinensetin; T—tangeretin; Sc—scutellarein tetramethylether.
